# Effect of glycated hemoglobin A1c on the survival of patients with oral squamous cell carcinoma: A multi-institutional database cohort study

**DOI:** 10.3389/fonc.2022.952616

**Published:** 2022-08-29

**Authors:** Chun-Yuan Chao, Sheng-Dean Luo, Wei-Chih Chen, Shao-Chun Wu, Tai-Jan Chiu, Yu-Ming Wang, Yao-Hsu Yang, Fu-Min Fang, Shau-Hsuan Li, Chung-Yi Li, Ching-Nung Wu

**Affiliations:** ^1^ Department of Otolaryngology, Kaohsiung Chang Gung Memorial Hospital and Chang Gung University College of Medicine, Kaohsiung, Taiwan; ^2^ Graduate Institute of Clinical Medical Sciences, College of Medicine, Chang Gung University, Taoyuan, Taiwan; ^3^ Department of Anesthesiology, Kaohsiung Chang Gung Memorial Hospital and Chang Gung University College of Medicine, Kaohsiung, Taiwan; ^4^ Department of Hematology-Oncology, Kaohsiung Chang Gung Memorial Hospital and Chang Gung University College of Medicine, Kaohsiung, Taiwan; ^5^ Department of Radiation Oncology, Kaohsiung Chang Gung Memorial hospital and Chang Gung University College of Medicine, Kaohsiung, Taiwan; ^6^ Department of Traditional Chinese Medicine, Chang Gung Memorial Hospital, Chiayi, Taiwan; ^7^ Health Information and Epidemiology Laboratory of Chang Gung Memorial Hospital, Chiayi, Taiwan; ^8^ School of Traditional Chinese Medicine, College of Medicine, Chang Gung University, Taoyuan, Taiwan; ^9^ Department of Public Health, College of Medicine, National Cheng Kung University, Tainan, Taiwan; ^10^ Department of Public Health, College of Public Health, China Medical University, Taichung, Taiwan; ^11^ Department of Healthcare Administration, College of Medical and Health Science, Asia University, Taichung, Taiwan

**Keywords:** oral squamous cell carcinoma (OSCC), diabetes mellitus, glycated hemoglobin A1c (HbA1C), all-cause mortality(ACM), disease-specific mortality, average real variability

## Abstract

**Objectives:**

Few studies have evaluated the impact of blood glucose levels on cancer prognosis. We investigated the association between hemoglobin A1c (HbA1c) and survival in oral squamous cell carcinoma (OSCC) patients.

**Materials and Methods:**

A 19-year retrospective cohort study of OSCC patients was performed using the Chang Gung Research Database to identify and enroll 7279 patients diagnosed with OSCC between January 2001 and June 2020. A total of 3600 patients were recruited after performing 1:2 frequency-matching between patients with DM and non-DM. A Cox’s regression model was used to evaluate the relative hazards of all-cause mortality (ACM) and disease-specific mortality (DSM) in relation to HbA1c.

**Results:**

An unadjusted Cox’s regression model indicated that DM, in addition to high levels of HbA1c, were statistically prognostic of poor survival. An adjusted hazard ratio (aHR) of HbA1c ≥ 8% interval at the initial diagnosis of OSCC was statistically higher for DSM (1.25 to 2.24) compared to the non-DM group in different regression models. Considering the effect of sustained HbA1c control in 699 patients, the aHR of mean HbA1c ≥ 9% interval was statistically higher for ACM (1.78 to 2.13) compared to the reference group (7% ≤ HbA1c< 8%). In addition, increased hazards of ACM (2.09 to 2.18) and DSM (2.20 to 2.41) were consistently observed in the highest quartiles of average real variability of HbA1c.

**Conclusion:**

Poor and unstable control of HbA1c could strongly predict the risks of mortality in OSCC patients with DM.

## Introduction

Oral squamous cell carcinoma (OSCC) is one of the most challenging problems worldwide. In 2018, more than 350,000 individuals worldwide were diagnosed with OSCC, and it was reported about 177,000 OSCC-related deaths (1). Patients with oral cancer have a higher comorbidity burden at diagnosis and survival outcome decreases significantly as the number of comorbidities increases (2). Diabetes mellitus (DM) is a global issue and worldwide prevalence is progressively increasing. A recent study reported 415 million diabetic patients in 2015 and 5 million deaths directly related to the disease, with a health cost of $673 billion (3). Previous studies have reported that patients with DM have a higher prevalence and chance of developing oral cancer than non-diabetic patients (4). Nevertheless, the effect of DM and glycated hemoglobin A1c (HbA1c) control on survival in oral cancer patients has not been studied fully.

There is a saying that glucose “nourishes” cancer and may therefore effect disease outcome in spite of the lack of evidence. The relationship between diabetes and cancer are not clearly explained by the biological mechanisms. Chronic hyperinsulinemia, caused by both endogenous insulin resistance and exogenous diabetic therapy, have been shown to promote malignant transformation through the activation of insulin-like growth factor (IGF) receptors (5, 6). On the other hand, because of cancer cell dependence on aerobic glycolysis required for generation of adenosine triphosphate (ATP), hyperglycemia has also been shown to increase the incidence of cancer and promote cancer growth (7). To date, very few clinical studies have evaluated the impact of blood glucose levels on cancer prognosis (8–10). Some studies have failed to adjust for cancer-related variables (such as staging and treatment), as well as accounting for factors related with the metabolic syndrome (including comorbidities and body mass index (BMI)), or medication use. Other studies failed to measure glucose or HbA1c levels, and those studies measuring HbA1c levels only assessed these data at the time of cancer diagnosis without monitoring the following changes to HbA1c levels. In addition, many previous studies analyzed the combined mortality from all cancers rather than the survival for oral cancer alone.

We therefore performed a study to evaluate the survival outcomes between patients with and without DM, with different HbA1c intervals at the time of cancer diagnosis and with different HbA1c intervals during follow-up examination. This study was completed using a population of oral cancer patients, using a retrospective cohort from a multi-institutional database.

## Methods

### Study cohort

**Figure 1:**
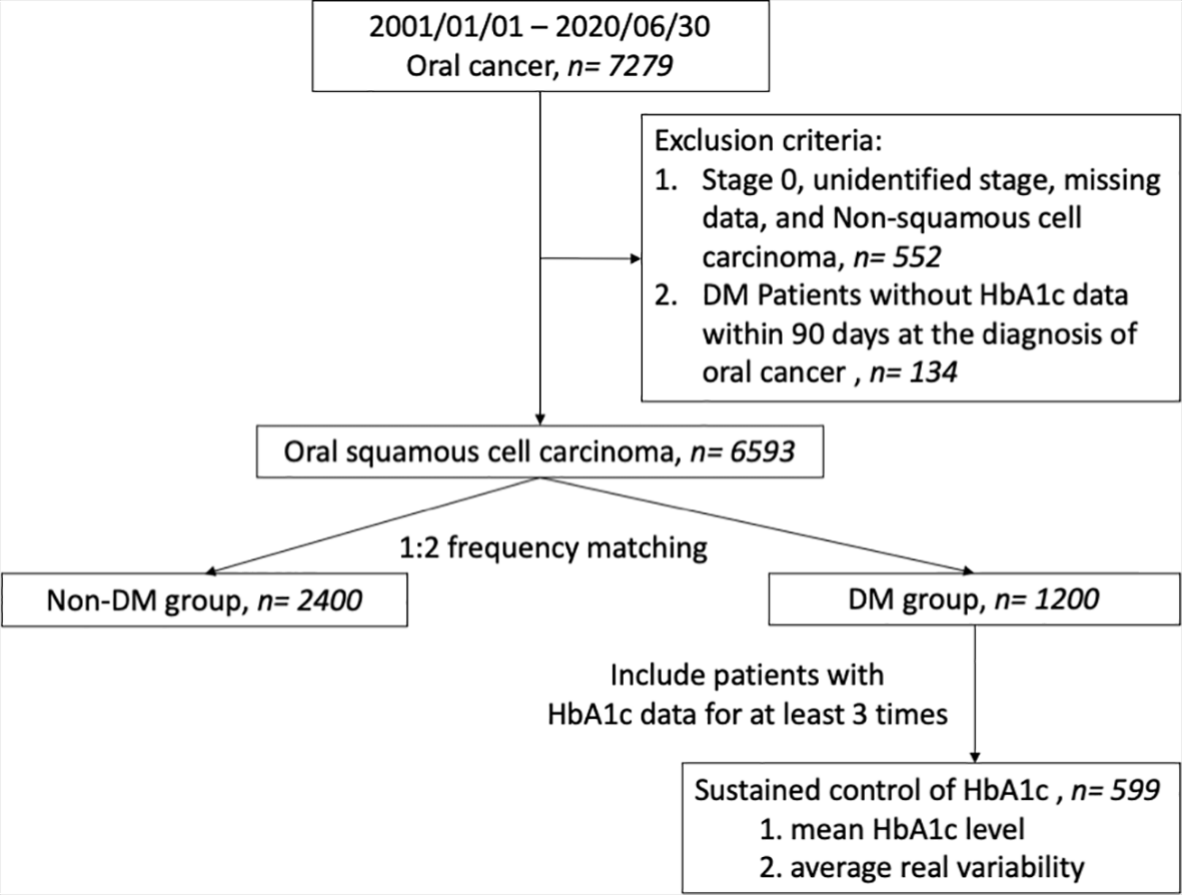
Flow diagram illustrating the cohort study design in patients with oral cancer. DM, diabetes mellitus.

A multicenter retrospective cohort study was performed using data from the Chang Gung Research Database, which was well validated in previous studies (11, 12). This study was approved by the Institutional Review Board of Kaohsiung branch of Chang Gung Memorial Hospital. **Figure 1** is a flow chart of the study design for statistical analysis. In all, 7279 oral cancer patients diagnosed between January 2001 and June 2020 whose information had been recorded into both the Chang Gung Research Database and Taiwan Cancer Registry were enrolled. Patients with either non-squamous cell carcinoma, an unclear tumor site or without staging data were excluded from the study. Patients with DM were identified by the following diagnostic codes: ICD-9-CM: 250.xx or ICD-10-CM: E08 to E13. The diagnostic codes were ensured to be present at least 3 times in outpatient records before a diagnosis of OSCC (13), or coexisting with an OSCC diagnosis. Patients diagnosed with DM, without HbA1c data within 90 days of a OSCC diagnosis were excluded. Therefore, a total of 6593 OSCC patients were recruited after applying the exclusion criteria (**Table S1**).

We performed frequency matching to control covariates of both DM and non-DM patients, including age at diagnosis, sex, tumor site, and clinical AJCC stage. Therefore, data from a total of 3600 patients were included, containing 1200 patients diagnosed with DM and 2400 matched patients without a DM diagnosis (**Table 1**). HbA1c levels were measured within 90 days of the initial OSCC diagnosis in DM patients and were categorized into groups (<6.0, 6.0-6.9, 7.0-7.9, 8.0-8.9, ≥9.0%; **Table S2**). The survival of these subgroups within DM patients were compared to the non-DM group. To evaluate if control of HbA1c levels is associated with survival, from the 1200 subjects we only included 699 patients who had HbA1c levels measured at least 3 times for further analysis. Mean HbA1c levels for each patient across the study period were calculated, and these patients were then categorized into five subgroups according to their mean HbA1c level (<6.0, 6.0≤ HbA1c<7.0, 7.0≤ HbA1c<8.0, 8.0≤ HbA1c<9.0, ≥9.0%) (**Table S3**). The survival of these subgroups was also compared.

**Table 1:** Demographic and clinical characteristics of OSCC patients after frequency matching.

Variables	OSCC patients n = 3600	Non-DM n = 2400	DM n = 1200	SMD
**Median age at diagnosis**,				0.029
years (IQR)	57 (50-64)	56 (50-64)	57 (50-64)	
**Gender**				—
Female Male	207 (05.8%) 3393 (94.2%)	138 (05.8%) 2262 (94.2%)	69 (05.8%) 1131 (94.2%)	
**Tumor sites**				—
Lip Oral tongue Upper/lower Gum Floor of mouth Buccal mucosa Hard palate Retromolar trigone	165 (04.6%) 1119 (31.1%) 648 (18.0%) 96 (02.7%) 1329 (36.9%) 54 (01.5%) 189 (05.2%)	110 (04.6%) 746 (31.1%) 432 (18.0%) 64 (02.7%) 886 (36.9%) 36 (01.5%) 126 (05.2%)	55 (04.6%) 373 (31.1%) 216 (18.0%) 32 (02.7%) 443 (36.9%) 18 (01.5%) 63 (05.2%)	
**Lifestyle Risk Factors**				
Smoking				0.158
No Yes	1649 (45.8%) 1951 (54.2%)	1162 (48.4%) 1238 (51.6%)	487 (40.6%) 713 (59.4%)	
Betel nuts consumption				0.151
No Yes	1840 (51.1%) 1760 (48.9%)	1287 (53.6%) 1113 (46.4%)	553 (46.1%) 647 (53.9%)	
Alcoholic beverages				0.220
No Yes	1226 (34.1%) 2374 (65.9%)	899 (37.5%) 1501 (62.5%)	327 (27.3%) 873 (72.7%)	
**Comorbidities**				
Hypertension				0.608
No Yes	3055 (84.9%) 545 (15.1%)	2219 (92.5%) 181 (07.5%)	836 (69.7%) 364 (30.3%)	
Dyslipidemia				0.621
No Yes	3202 (88.9%) 398 (11.1%)	2302 (95.9%) 98 (04.1%)	900 (75.0%) 300 (25.0%)	
**Clinical AJCC staging**				—
I II III IVa IVb IVc	696 (19.3%) 744 (20.7%) 471 (13.1%) 1254 (34.8%) 420 (11.7%) 15 (00.4%)	464 (19.3%) 496 (20.7%) 314 (13.1%) 836 (34.8%) 280 (11.7%) 10 (00.4%)	232 (19.3%) 248 (20.7%) 157 (13.1%) 418 (34.8%) 140 (11.7%) 5 (00.4%)	
**Pathological AJCC staging**				0.080
I II III IVa IVb	731 (20.3%) 683 (19.0%) 455 (12.6%) 1115 (31.0%) 256 (07.1%)	466 (19.4%) 463 (19.3%) 316 (13.2%) 747 (31.1%) 167 (07.0%)	265 (22.1%) 220 (18.3%) 139 (11.6%) 368 (30.6%) 89 (07.4%)	
**Treatment**				0.012
Operation alone Operation plus RT/CCRT RT/CCRT Others	1816 (50.4%) 1493 (41.5%) 184 (05.1%) 107 (03.0%)	1215 (50.6%) 991 (41.3%) 123 (05.1%) 71 (03.0%)	601 (50.1%) 502 (41.8%) 61 (05.1%) 36 (03.0%)	
**BMI** (IQR)	24.6 (21.9-27.3)	24.1 (21.6-26.7)	25.3 (22.9-28.1)	0.330
**Lab data** (IQR)				
HbA1C Total cholesterol	6.6 (5.9-8.0) 181 (155-207.5)	5.8 (5.6-6.1) 183 (158-208)	7.7 (6.8-9.4) 175 (149-207)	1.713 0.078
**Medication**				
Statins				0.561
No Yes	3098 (86.1%) 502 (13.9%)	2229 (92.9%) 171 (07.1%)	869 (72.4%) 331 (27.6%)	
Metformin				1.098
No Yes	2948 (81.9%) 652 (18.1%)	2299 (95.8%) 101 (04.2%)	649 (54.1%) 551 (45.9%)	

AJCC, American Joint Committee on Cancer; BMI, body mass index; CCRT, concurrent chemoradiotherapy; DM, diabetes mellitus; IQR, interquartile range; OSCC, oral squamous cell carcinoma; RT, radiotherapy; SMD, standardized mean difference.

In addition, the variability of HbA1c levels was a concern and so the average real variability (ARV) was calculated in the 699 patients. The ARV is an indicator that reflects the sum of variability between two successive measurements within a subject and is known to be a more reliable representation of variability than the standard deviation. It was calculated as the average of the absolute differences between consecutive HbA1c measurements, which the formula is shown as below:


$$ARV=\frac{1}{n-1}\sum_{k=1}^{n}\left|HbA1c_{k+1}-HbA1c_{k}\right|$$

where n is the total number of HbA1c measurement within a subject, and k is the ordinal number of HbA1c measurement within a subject. For example, if a patient had four HbA1c measurement during follow up, e.g., 6.5, 7.5, 9, 8.5, then ARV = [(|7.5-6.5|) + (|9-7.5|) + (|8.5-9|)]/3 = 1. These patients were then further stratified into four groups by quartiles for survival analysis. The lowest quartile of ARV (quartile 1) referred to the minimal absolute differences between consecutive HbA1c measurements; the highest quartile (quartile 4) referred to the maximum absolute differences.

### Statistical analysis

The demographic and clinical characteristics of the patients were summarized using descriptive statistics. Standardized mean difference (SMD) was calculated between the characteristics of two groups, and a significant between-group difference was found when SMD > 0.1 (14). Parametric continuous data was analyzed using Student’s t-test and non-parametric data were analyzed by the Mann–Whitney U test, respectively. Categorical data (such as sex, tumor site, lifestyle risk factors, comorbidities, clinical and pathological AJCC stage, treatment modalities, and medication use) were analyzed by a two-sided Pearson’s chi-square test or a two-sided Fisher’s exact test. To make the two groups more comparable in the baseline characteristics, data were analyzed from a 1:2 frequency matching cohort (DM vs. non-DM). The Kaplan–Meier method and log-rank test were used to evaluate the effect of different HbA1c levels on overall survival (OS) and disease-specific survival (DSS). The Cox proportional hazards model was used to test the crude effect and the adjusted effect in multivariate survival modeling. Several adjusted models were constructed and tested as a sensitivity analysis. Statistical analyses were done using SAS software (SAS Institute Inc., Cary, NC, USA), version 9.4 of the SAS System for Windows. Statistically significant were considered when *p*-values< 0.05.

## Results

Among the 7,279 patients with oral cancer, a total of 6,593 OSCC patients were recruited after applying the exclusion criteria (**Table S1**). Many factors including, age, tumor site, and AJCC staging, which affect cancer survival, varied between patients with and without DM. In addition, lifestyle risk factors, BMI, comorbidities, and medications for chronic health problems were also significantly different between the two groups.

A total of 3,600 patients remained after performing 1:2 frequency matching to control for factors known to effect cancer survival. These included: age at diagnosis, sex, tumor site, and clinical AJCC stage. In all, 1,200 were identified as patients with DM and 2,400 as non-DM (**Table 1**). There was a higher number of DM patients with more lifestyle risk factors, such as smoking, betel nut consumption, and alcohol consumption. In addition, BMI, prevalence of comorbidities, and use of medications for chronic disease were also higher in DM patients. Nevertheless, there was no difference between cancer staging, treatment and time from diagnosis to surgery when comparing the two groups, after matching. With regards to the impact of prognostic factors on survival, Cox regression analysis suggested that various factors, such as age, BMI, hypertension, DM, level of total cholesterol, tumor site, clinical and pathological AJCC stages of cancer, treatment, and tumor recurrence were associated with OS significantly. On the other hand, the abovementioned variables in addition to the levels of HbA1c correlated with DSS, excluding age and hypertension (**Table 2**).

**Table 2:** Univariate analyses of prognostic factors for all-cause mortality and disease-specific mortality in patients with oral cancer.

Factor	All-cause mortalityHazard ratio (95% CI) *p* value		Disease-specific mortality Hazard ratio (95% CI) *p* value	
**Age (year (IQR))**	1.019 (1.014-1.024)	*<0.001	1.004 (0.997-1.010)	0.244
**Gender**				
Male Female	1 1.18 (0.96-1.44)	0.117	1 1.03 (0.78-1.36)	0.821
**Tumor sites**				
Lip	1		1	
Oral tongue Upper/lower Gum Floor of mouth Buccal mucosa Hard palate Retromolar trigone	1.59 (1.21-2.11) 2.02 (1.52-2.68) 1.16 (0.76-1.76) 1.30 (0.98-1.71) 2.67 (1.73-4.11) 1.65 (1.18-2.31)	*0.001 *<0.001 0.495 0.068 *<0.001 *0.004	1.37 (0.96-1.95) 1.90 (1.33-2.73) 0.90 (0.51-1.59) 1.18 (0.83-1.68) 1.87 (1.04-3.38) 1.56 (1.02-2.38)	0.080 *<0.001 0.723 0.351 *0.038 *0.041
**Lifestyle Risk Factors**				
Smoking		0.079		0.447
No Yes	1 0.91 (0.82-1.01)		1 0.95 (0.83-1.09)	
Betel nuts consumption		0.121		0.843
No Yes	1 0.92 (0.83-1.02)		1 1.01 (0.89-1.16)	
Alcoholic beverages		0.053		0.544
No Yes	1 0.90 (0.81-1.00)		1 0.96 (0.84-1.10)	
**Comorbidities**				
Hypertension		*<0.001		0.499
No Yes	1 1.25 (1.10-1.43)		1 1.06 (0.89-1.28)	
Diabetes mellitus		*0.005		*0.004
No Yes	1 1.16 (1.05-1.29)		1 1.22 (1.07-1.40)	
Dyslipidemia		0.975		0.060
No Yes	1 1.00 (0.85-1.17)		1 0.81 (0.65-1.01)	
**Clinical AJCC staging**				
I	1		1	
II III IVa IVb IVc	1.19 (0.99-1.44) 1.50 (1.23-1.84) 2.60 (2.22-3.06) 4.30 (3.57-5.17) 17.37 (10.25-29.46)	0.068 *<0.001 *<0.001 *<0.001 *<0.001	1.26 (0.95-1.68) 1.91 (1.42-2.56) 3.77 (2.98-4.78) 7.19 (5.56-9.28) 34.99 (20.11-60.86)	0.112 *<0.001 *<0.001 *<0.001 *<0.001
**Pathological AJCC staging**				
I	1		1	
II III IVa IVb	1.23 (1.00-1.50) 1.75 (1.42-2.15) 3.06 (2.58-3.62) 4.64 (3.73-5.79)	*0.048 *<0.001 *<0.001 *<0.001	1.28 (0.94-1.75) 1.92 (1.40-2.63) 4.61 (3.60-5.92) 6.72 (4.96-9.10)	0.120 *<0.001 *<0.001 *<0.001
**Treatment**				
Operation alone	1		1	
Operation plus RT/CCRT RT/CCRT Others	1.76 (1.58-1.96) 5.09 (4.25-6.09) 10.59 (8.50-13.21)	*<0.001 *<0.001 *<0.001	2.29 (1.97-2.66) 7.21 (5.79-8.99) 14.96 (11.51-19.44)	*<0.001 *<0.001 *<0.001
**BMI** (IQR)	0.94 (0.92-0.95)	*<0.001	0.94 (0.92-0.96)	*<0.001
**Lab data** (IQR)				
HbA1C Total cholesterol	1.02 (0.99-1.06) 0.995 (0.993-0.996)	0.213 *<0.001	1.05 (1.01-1.10) 0.994 (0.992-0.996)	*0.014 *<0.001
**Recurrence**		*<0.001		*<0.001
No Yes	1 3.20 (2.89-3.54)		1 5.46 (4.79-6.22)	

*p ≤ 0.05.

AJCC, American Joint Committee on Cancer; BMI, body mass index; CCRT, concurrent chemoradiotherapy; IQR, interquartile range; RT, radiotherapy.

**Figure 2:**
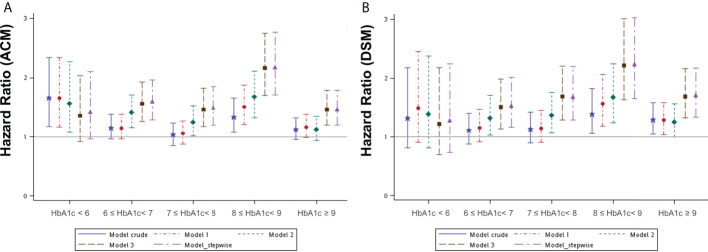
Hazard ratios for mortality events according to the different HbA1c intervals at the initial diagnosis of OSCC in patients with DM compared to patients without DM by several models. **(A)** ACM, all-cause mortality; **(B)** DSM, disease-specific mortality. DM, diabetes mellitus. OSCC, oral squamous cell carcinoma Model 1 was adjusted for age, sex, tumor site, and clinical AJCC stages of cancer. Model 2 was adjusted for the variables in model 1 plus BMI, lifestyle risk factors, and treatment. Model 3 was adjusted for the variables in model 2 plus comorbidities and medication use. Model 4_ stepwise was built with variables according to the statistical software (a stepwise solution).

To further investigate the effect of HbA1c levels on survival, DM patients were divided into five subgroups according to the level of HbA1c measured within 90 days of the initial OSCC diagnosis (**Table S2**). A Kaplan–Meier survival analysis was performed to evaluate whether different HbA1c intervals in DM patients accounted for different survival outcomes compared to patients without DM in a OSCC population (**Figures S1A, B**). This revealed a statistically significant difference in both OS and DSS between these subgroups. Several Cox regression models were built to assess the effect of different HbA1c intervals at the initial diagnosis of OSCC on all-cause mortality (ACM) and disease-specific mortality (DSM) compared to those without DM (**Table S4**). The crude model determined the survival effect of different HbA1c intervals compared to the non-DM group, without adjustment. Model 1 was adjusted for the variables known to affect cancer survival, including age, sex, tumor site, and clinical AJCC staging. Model 2 was adjusted for the variables in model 1 plus BMI, lifestyle risk factors (smoking, betel nut consumption, and alcohol consumption), and treatment. Model 3 was adjusted for all potential confounding variables, including those adjusted for in Model 2 plus comorbidities (hypertension and dyslipidemia) and medication use (metformin and statins). Model 4 was constructed using the stepwise solution presented in the statistical software. The hazard ratio (HR) was estimated to be 1.06 to 2.17 for ACM after adjustment for different HbA1c intervals compared to the non-DM group. The HR was statistically higher in the 8.0-8.9% interval. As for DSM, the HR was 1.15 to 2.24 after adjustment for different HbA1c intervals compared to the non-DM group, and it remained statistically higher in the 8.0-8.9 and ≥9.0% intervals (**Figures 2A, B**). The numerical results of the associations between HbA1c intervals of the initial OSCC diagnosis and both ACM and DSM corresponding to **Figure 2** are presented in **Table S4**.

**Figure 3:**
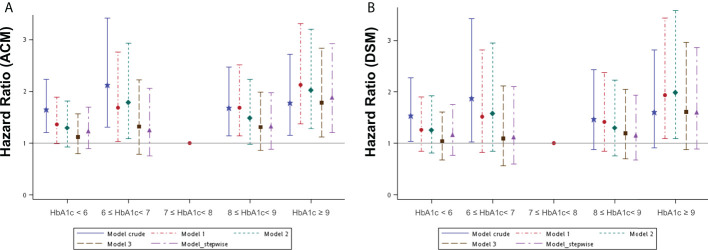
Hazard ratios for mortality events according to the different mean HbA1c intervals during the whole study period in patients with diabetes mellitus by several models. **(A)** ACM, all-cause mortality; **(B)** DSM, disease-specific mortality. Model 1 was adjusted for age, sex, tumor site, and clinical AJCC stages of cancer. Model 2 was adjusted for the variables in model 1 plus BMI, lifestyle risk factors, and treatment. Model 3 was adjusted for the variables in model 2 plus comorbidities and medication use. Model 4_ stepwise was built with variables according to the statistical software (a stepwise solution).

To understand if the control of HbA1c levels is associated with survival, we selected 699 patients with HbA1c data available for at least three separate occasions for further analysis. The mean HbA1c data for each patient from across the entire study period was calculated and these patients were then categorized into five subgroups according to their mean HbA1c level (**Table S3**). A Kaplan–Meier survival analysis was performed to evaluate whether different mean HbA1c levels affected survival outcomes in an OSCC population (**Figures S2A, B**). This analysis revealed a statistically significant difference only in OS between these subgroups. Several Cox regression models were built to assess the effect of different mean HbA1c intervals across the whole study period on ACM and DSM (**Table S5**). The crude model determined the survival effect of different mean HbA1c intervals, with a control reference to the survival of 7≤ HbA1c< 8% interval. Other adjusted models were built in the same way as **Table S4** describes. The HR was 1.12 to 2.13 for ACM after adjustment in different mean HbA1c intervals compared to the reference interval (7≤ HbA1c< 8%). It remained statistically higher in the HbA1c ≥ 9% interval with an effect size between 1.78 and 2.13 in different models. As for DSM, it was 1.04 to 1.98 after adjustment in different mean HbA1c intervals compared to the reference interval, and the effect size of the HbA1c ≥ 9% interval in different models was between 1.60 and 1.98, although not statistically significant consistently (**Figures 3A, B**). The numerical results of the associations between mean HbA1c intervals across the entire study period and both ACM and DSM corresponding to **Figure 3** are presented in **Table S5**.

The variability of HbA1c levels was another factor to be considered when assessing the control of diabetes, and ARV was calculated for the 699 patients. The ARV data were stratified into four groups by quartiles and the HR for ACM and DSM was calculated in different quartiles and compared to the lowest quartile (**Table 3**). In the crude model, the highest quartile of ARV showed an HR of 1.89 and 2.18 for ACM and DSM, respectively. Other adjusted models were built as **Table S4** describes. All of the HR values in the highest quartile of ARV were consistently increased for both ACM and DSM after adjustment in different models compared to the lowest quartile of ARV.

**Table 3:** Modeling for the effects of ARV on all-cause mortality and disease-specific mortality in OSCC patients with DM .

Outcomes	**ARVquartiles	Crude Hazard Ratio (95% CI)	Adjusted Hazard Ratio (95% CI)			
			^¶^Model 1	^§^Model 2	^□^Model 3	^❡^Model 4
**All-cause mortality**	1 (ARV<0.42) 2 (0.42≤ ARV< 0.65) 3 (0.65≤ ARV< 1.05) 4 (ARV≥1.05)	1 0.87 (0.61-1.23) 0.99 (0.70-1.41) *1.89 (1.37-2.60)	1 1.04 (0.72-1.48) 1.18 (0.83-1.70) *2.10 (1.51-2.94)	1 1.06 (0.73-1.53) 1.24 (0.85-1.80) *2.18 (1.54-3.08)	1 0.95 (0.66-1.38) 1.16 (0.79-1.68) *2.09 (1.47-2.98)	1 0.95 (0.67-1.35) 1.08 (0.75-1.55) *2.14 (1.52-3.01)
**disease-specific mortality**	1 (ARV<0.42) 2 (0.42≤ ARV< 0.65) 3 (0.65≤ ARV< 1.05) 4 (ARV≥1.05)	1 1.12 (0.70-1.78) 1.25 (0.79-1.99) *2.18 (1.41-3.35)	1 1.27 (0.79-2.05) 1.40 (0.86-2.26) *2.21 (1.41-3.46)	1 1.31 (0.80-2.15) 1.57 (0.96-2.58) *2.41 (1.51-3.83)	1 1.29 (0.79-2.11) 1.54 (0.94-2.53) *2.40 (1.49-3.87)	1 1.20 (0.75-1.92) 1.34 (0.83-2.16) *2.20 (1.40-3.46)

*p ≤ 0.05.

ARV, average real variability; CI, confidence interval; DM, diabetes mellitus; OSCC, oral squamous cell carcinoma.

**Quartile 1 refers to the minimal absolute differences between consecutive HbA1c measurements; quartile 4 refers to the maximal absolute differences between consecutive HbA1c measurements.

^¶^Model 1 was adjusted for age, sex, tumor site, and clinical AJCC stages of cancer.

^§^Model 2 was adjusted for the variables adjusted in model 1 plus BMI, lifestyle risk factors, and treatment.

^□^Model 3 was adjusted for the variables adjusted in model 2 plus comorbidities and medication use.

^❡^Model 4 was built with variables according to the statistical software (a stepwise solution).

## Discussion

To the best of our knowledge, this is the first study to investigate the effect of HbA1c level on survival outcome in OSCC patients. In this 19-year retrospective cohort study, the presence of DM in addition to higher levels of HbA1c at initial OSCC diagnosis correlated with statistically higher DSM. Poor and unstable control of HbA1c levels during follow up could be a strong predictor of the risks for mortality in OSCC patients with DM.

It has been shown that OSCC patients have a significantly higher comorbidity burden at diagnosis and that survival outcomes are significantly decreased with the presence of additional comorbidities (2, 15, 16). Several hypotheses concerning the underlying biological mechanisms have been suggested for the reduced survival in patients with malignancies. First, it is postulated that DM associated with hyperinsulinemia may lead to an increase in tumor cell proliferation and metastases in cancer patients. A previous study showed that high levels of insulin or a followed-by increase in IGF‐1 may promote cancer growth (5, 17). Many theories on cancer energetics and the Warburg hypothesis as well often emphasize the dependence of cancers on glycolysis (7, 18). Finally, adipose tissue has been described as an active endocrine organ that produces a variety of factors such as monocyte chemoattractant protein, interleukin-6, tumor necrosis factor-α, free fatty acids, adiponectin, and leptin, which might be associated with the regulation of malignant transformation or cancer progression (19). Those hypotheses partly explains why the patients with type 2 DM had an increased risk for some cancers and why DM itself can be a risk factor for cancer-specific mortality in multiple studies, including this one (20).

In our study, we found that patients with DM significantly suffered from increased ACM (HR: 1.16 [95% CI 1.10 –1.43]) and DSM (HR: 1.22 [95% CI 1.07 –1.40]) in univariate analysis. There are several clinical explanations for the observed association between increased ACM and DSM in OSCC patients with DM. First, diabetic cancer patients are frequently treated less aggressively and therefore have a worse prognosis compared to those without diabetes (21). Second, more prone to postoperative complications or limited access to adjuvant therapies was noted in patients with diabetes compared to their nondiabetic counterparts (22). Lastly, cardiovascular morbidities and metabolic syndromes associated with DM may increase noncancer death after operation. It could be another reason explaining the risk for all-cause mortality in OSCC patients with DM (10). Our study result was consistent with the abovementioned theories. In our study, DM patients in the subgroup of HbA1c< 6% interval had the worst survival rate in our crude model, but the effect became less visible after adjusting other variables (**Table S5**). A possible explanation is that a cancer-related change in diet would result in a malnutritioned status complicated further by poor control of blood sugar. In another words, other cancer-related factors, such as tumor site, cancer stage, and the way of treatment, might be correlated with the malnutritioned status and result in poor sugar control. Besides, the cancer-related factors were strongly associated with the survival in OSCC patients. In brief, those factors confound the effect of blood sugar control on the survival of DM patients with OSCC, and therefore, should be adjusted in regression models. The significant effect leading to higher mortality remained in the subgroup of HbA1c ≥ 9 after adjusting other possible confounding, which echoed that nutritional management and proper blood sugar control may become an integral component of head and neck cancer management (23).

It is suggested that glycemic metabolism is altered in head and neck cancer according to recent publications (24). Elevated blood glucose levels around the time of cancer diagnosis correlated with reduced survival rates in head and neck cancer. In addition, the model of insulin resistance has been associated with disease-free survival independently, indicating that the prognosis in this group of patients might get better after improving glycemic control (24, 25). Our study supports these opinions and reveals that patients with diabetes and high levels of HbA1c are statistically associated with higher ACM and DSM. However, this conclusion is not in line with previous reports. Boursi et al. found that there was no association between HbA1C levels and survival among patients with cancer and concurrent DM (9). Two limitations are associated with that study. First, specific cancer risk factors, such as staging and treatment, were not adjusted. Second, continuous HbA1c data were used to compute the HR, which assumes linearity. Accordingly, low and high mean HbA1c values were associated with increased ACM, which showed a U-shaped HR plot (26). Therefore, the survival effect of HbA1c levels would be offset unless the continuous HbA1c data were categorized. Our result is generally in accordance with the U-shaped association. We have shown that a mean HbA1c interval between 7 and 8% was associated with lowest ACM and DSM in OSCC patients, which was consistent with previous literature (26). An increase or decrease from this mean HbA1c interval was associated with heightened risk of adverse outcomes. The U-shaped pattern of risk association was sufficiently similar, though becoming less apparent after adjustment, to suggest that risk of mortality with respect to HbA1c was independent of other factors. Worse ACM in patients achieving low mean percentages of HbA1c might be related to hypoglycemia, it is a common complication of intensive blood-glucose control. Hypoglycemia is associated with various sequelae that could increase mortality. For instance, a link exists between the sympathomimetic (adrenergic) or hypokalemic manifestations of hypoglycemia may onset of cardiac arrhythmia, this might predispose patients who already have underlying cardiovascular disease to have more atherosclerotic plaque formation and cause vascular dysfunction (27). Thus less stringent targets might be appropriate for patients with more advanced disease of longer duration and higher baseline HbA1c concentration.

Several regression models were used to estimate the real effect of blood sugar control on the survival of DM patients with OSCC, and the effect size seems to be closer to the null after adjusting more variables from the model 1 to the model 3. The model 4 was another way of regression based on the stepwise solution in the statistical software. The effect size seemed to be similar in the model 3 and the model 4 in either ACM or DSM, indicating the effects found in the model 3 and the model 4 could be deemed as real associations between different intervals of HbA1c control and mortality. On the other hand, glycemic variability has attracted a lot of attention recently as an important component of blood sugar control. Several studies also provided evidence on the relationship between glycemic variability and diabetes-related outcomes (28). Our study also supported those findings, which both HbA1c ≥ 9% and the highest quartile of ARV showed significant impact on ACM and DSM of OSCC patients in our adjusted regression models (**Table S5** and **Table 3**). However, it is not known whether high HbA1c level or high glycemic variability has more impact on the survival. Lu, et al. (29) found that the association of HbA1c with all-cause mortality seemed to be weakened in individuals with the highest tertile of glucose variability, implying that patients with a high degree of glycemic variability has more impact on mortality than high level of HbA1c. In our study, the highest quartile of ARV had higher adjusted hazard ratio in both ACM and DSM across several models when compared to HbA1c ≥ 9%, which indicated that glycemic variability had greater impact in our study. However, the reference to be compared was different in the two groups, and therefore, the conclusion for this issue should be conservative.

There were several issues that readers might concern in this study. First, the ratio of advanced stages seems to be high (stage III: 13.1%, stage IV: 46.9%) based on general epidemiology. According to the CANCER REGISTRY ANNUAL REPORT (2019, TAIWAN), 12.1% were at stage III, and 43.0% were at stage IV among medical centers after excluding patients with stage 0 and unknow staging (30). The Chang Gung medical system served as tertiary care in Taiwan, and therefore the ratio of advanced stages was high but compatible to the data among medical centers in Taiwan. Second, although the way of treatment between the two groups were similar after matching, some might consider that less ratio of chemotherapy uses owing to a possible decreased renal function in DM group contributed the survival difference between the two groups. In our cohort, the ratio of cisplatin use was 721/2400 = 30.0% in the non-DM group, and 336/1200 = 28.0% in the DM group respectively. When considering the use of carboplatin in addition, the ratio of cisplatin or carboplatin use was 741/2400 = 30.9% in the non-DM group, and 355/1200 = 29.6% in the DM group respectively. There was no statistically significant difference in the use of chemotherapy between the two groups (*p*=0.4272). Lastly, HbA1c measurement was sensitive to the transfusion of red blood cell (RBC). There was 54 of 1200 patients, who had received RBC transfusion within 1 week before measuring HbA1c in the DM group. The percentage was only 4.5%. In addition, although the potential effect of transfusion on HbA1c has been recognized for some time, opinions on the direction of the effect are contradictory (31). Due to the abovementioned reasons, the effect of blood transfusion in our final result might be ignored.

Previous studies measured HbA1c level only at the time of cancer diagnosis without considering the following changes in HbA1c, unlike in this study. This can be considered a limitation when studying patients with oral cancer. Malnutrition complicated with unstable blood sugar control could be identified before diagnosis of oral cancer, which would deem the HbA1c level at the time of cancer diagnosis unrepresentative. Considering this limitation, this study measured the mean HbA1c data and HbA1c variability during follow-up to further clarify if survival outcome was affected by blood sugar control. Mean HbA1c level ≥ 9% and the highest quartile of ARV predicted higher mortalities, consistent with previous reports (32). Nevertheless, our study did have a number of limitations. First, the data were obtained from a de-identified research database and some clinical features, such as type 1 or type 2 DM, were unable to be distinguished. Second, the Chang Gung medical system served as tertiary care in Taiwan. Patients diagnosed with DM were often treated in a primary care system at first, before being subsequently referred to the Chang Gung medical system for further management of suspected head and neck cancer. Therefore, the impact of diabetes, time, and control before OSCC diagnosis could not be assessed. Third, the staging system was not consistent in this cohort because the codings changed from AJCC 6th edition (before 2010) to AJCC 7th edition (2010–2018) and AJCC 8th edition (after 2018). Finally, residual or unknown confounding variables are still possible after adjusting for the most relevant confounding factors. Despite these limitations, our results have important clinical implications for managing OSCC patients with DM. This prompts a need for a well-planned interventional study to be conducted to confirm these findings.

## Conclusion

This is the first study to investigate the effect of HbA1c levels on the survival of patients with OSCC. Patients with DM exhibited poor OS and DSS. In addition, mean HbA1c levels of ≥ 9% and a high variability of HbA1c during follow up significantly increased ACM and DSM. This study highlights the survival benefit of adequate and stable control of blood sugar levels in an OSCC population

## Author’s declaration

This paper had been disclosed in the conference “The Lancet Summit—Cancer care in Asia and Latin America” in July 2022. The abstract was modified and recruited in the Abstract booklet.

## Data availability statement

The data analyzed in this study is subject to the following licenses/restrictions: The data cannot be shared publicly because it is owned by Chang Gung Medical Branches and authors do not have permission to share the data. Data are available from the Department of Medical Research and Development for researchers who meet the criteria for access to confidential data. Requests to access these datasets should be directed to Ms. Shu-Jyuan Chiou, taytay@cgmh.org.tw;.

## Ethics statement

The studies involving human participants were reviewed and approved by Institutional Review Board of Kaohsiung branch of Chang Gung Memorial Hospital (IRB No. 202001135B0C601, IRB no. 202200213B0). Written informed consent for participation was not required for this study in accordance with the national legislation and the institutional requirements.

## Author contributions

Conceptualization, Writing - Original Draft: C-YC; Writing - Review & Editing, Supervision: S-DL; Investigation: W-CC; Software, Formal analysis: S-CW; Visualization: T-JC; Visualization: Y-MW; Formal analysis, Validation: Y-HY; Data Curation: F-MF; Resources: S-HL; Methodology: C-YL, Project administration, Funding acquisition, Conceptualization: C-NW. All authors contributed to the article and approved the submitted version.

## Funding

This work was supported by Kaohsiung Chang Gung Memorial Hospital, Taiwan, CFRPG8K0131 and CORPG8L0481. The funders had no role in study design, data collection and analysis, decision to publish, or preparation of the manuscript.

## Acknowledgments

We appreciate the Biostatistics Center at Kaohsiung Chang Gung Memorial Hospital and the Health Information and Epidemiology Laboratory at the Chiayi Chang Gung Memorial Hospital for helping with the study design and statistics work.

## Conflict of interest

The authors declare that the research was conducted in the absence of any commercial or financial relationships that could be construed as a potential conflict of interest.

## Publisher’s note

All claims expressed in this article are solely those of the authors and do not necessarily represent those of their affiliated organizations, or those of the publisher, the editors and the reviewers. Any product that may be evaluated in this article, or claim that may be made by its manufacturer, is not guaranteed or endorsed by the publisher.

## Supplementary material

Supplementary Figure 1 Kaplan–Meier survival curve for survival rates of different HbA1c intervals at the initial diagnosis of OSCC in patients with DM and without DM. **(A)** Overall survival; **(B)** Disease-specific survival. DM, diabetes mellitus. OSCC, oral squamous cell carcinoma.

Supplementary Figure 2 Kaplan–Meier survival curve for survival rates of different mean HbA1c levels during the whole study period in patients with diabetes mellitus. **(A)** Overall survival; **(B)** Disease-specific survival.
