# Supplementary figures and images for: Effect of glycated hemoglobin A1c on the survival of patients with oral squamous cell carcinoma: A multi-institutional database cohort study
Source: Front Oncol. 2022 Aug 29;12:952616. doi: 10.3389/fonc.2022.952616 (PMC9465414; doi:10.3389/fonc.2022.952616)

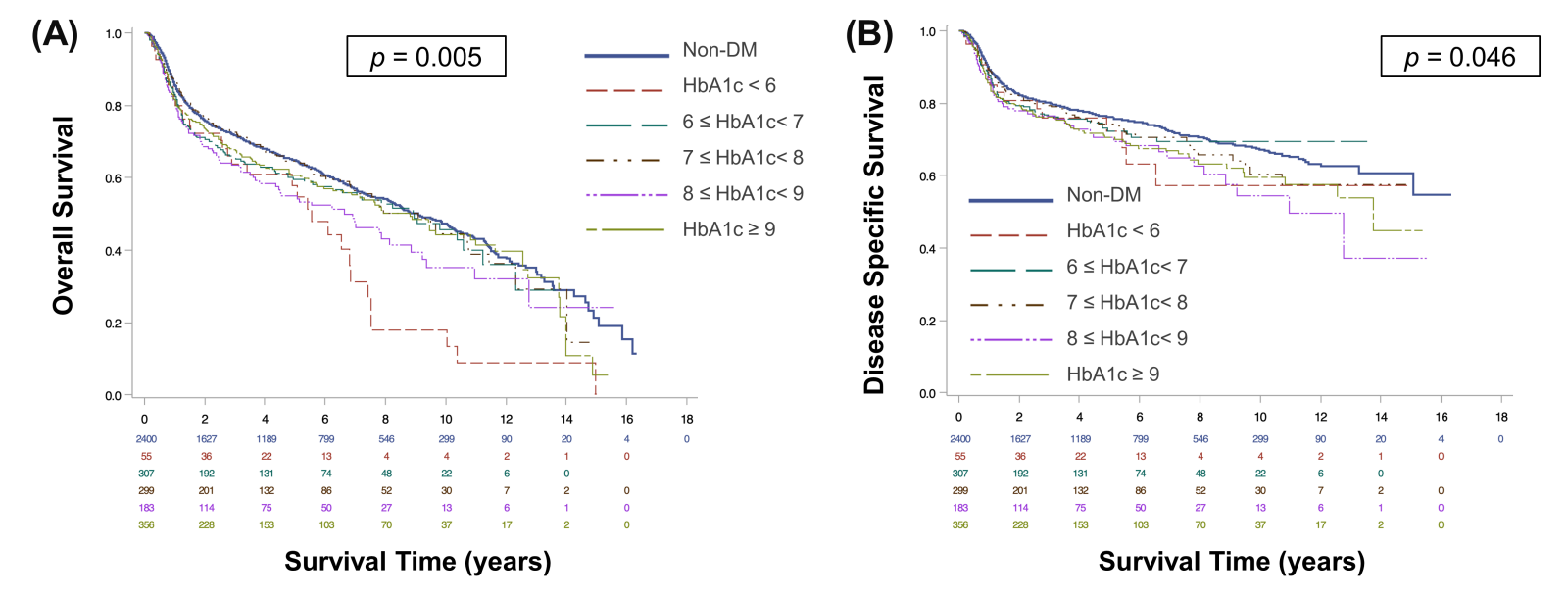

Supplement: Supplementary Figure 1 — Kaplan–Meier survival curve for survival rates of different HbA1c intervals at the initial diagnosis of OSCC in patients with DM and without DM. (A) Overall survival; (B) Disease-specific survival. DM, diabetes mellitus. OSCC, oral squamous cell carcinoma. [file Image_1.png]

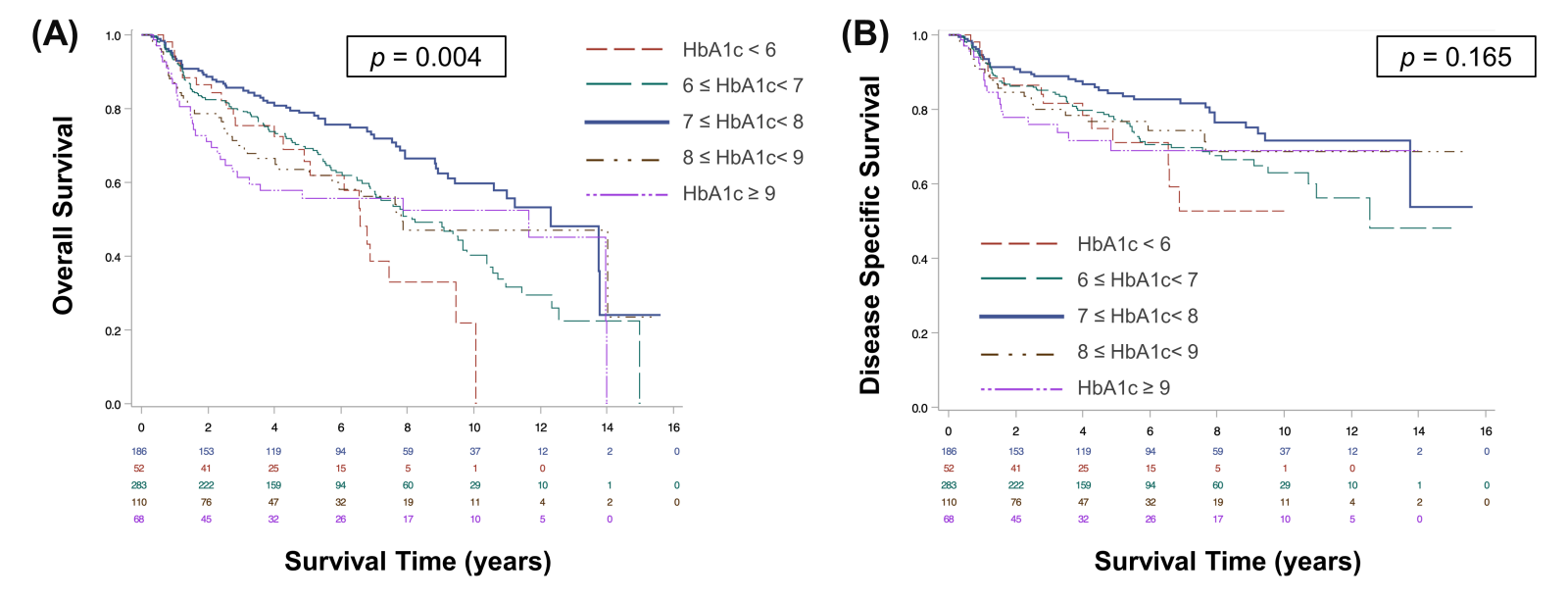

Supplement: Supplementary Figure 2 — Kaplan–Meier survival curve for survival rates of different mean HbA1c levels during the whole study period in patients with diabetes mellitus. (A) Overall survival; (B) Disease-specific survival. [file Image_2.png]
