# Supplementary material for: Effect of glycated hemoglobin A1c on the survival of patients with oral squamous cell carcinoma: A multi-institutional database cohort study
Source: Front Oncol. 2022 Aug 29;12:952616. doi: 10.3389/fonc.2022.952616 (PMC9465414; doi:10.3389/fonc.2022.952616)
Supplement: Supplementary file 3 [file Table_1.docx]

**Table S1.** Demographic and clinical characteristics of OSCC patients before matching

| **Variables** | **OSCC patients**  **n=6593** |  | **Non-DM**  **n =5267** | **DM**  **n =1326** | **SMD** |
| --- | --- | --- | --- | --- | --- |
| **Median age at diagnosis**,  years (IQR) | 54(46-62) |  | 53(45-61) | 58(51-65) | 0.446 |
| **Gender**  Female  Male | 587(08.9%)  6006(91.1%) |  | 482(09.2%)  4785(90.8%) | 105(07.9%)  1221(92.1%) | 0.044 |
| **Tumor sites**  Lip  Oral tongue  Upper/lower Gum  Floor of mouth  Buccal mucosa  Hard palate  Retromolar trigone | 317(04.8%)  2337(35.4%)  973(14.8%)  296(04.5%)  2139(32.5%)  167(02.5%)  364(05.5%) |  | 242(04.6%)  1947(37.0%)  735(14.0%)  254(04.8%)  1661(31.5%)  141(02.7%)  287(05.4%) | 75(05.6%)  390(29.4%)  238(18.0%)  42(03.2%)  478(36.0%)  26(02.0%)  77(05.8%) | 0.213 |
| **Lifestyle Risk Factors** |  |  |  |  |  |
| Smoking  No  Yes | 3207(48.6%)  3386(51.4%) |  | 2654(50.4%)  2613(49.6%) | 553(41.7%)  773(58.3%) | 0.175 |
| Betel nuts consumption  No  Yes | 3588(54.4%)  3005(45.6%) |  | 2961(56.2%)  2306(43.8%) | 627(47.3%)  699(52.7%) | 0.179 |
| Alcoholic beverages  No  Yes | 2414(36.6%)  4179(63.4%) |  | 2053(39.0%)  3214(61.0%) | 361(27.2%)  965(72.8%) | 0.252 |
| **Comorbidities** |  |  |  |  |  |
| Hypertension  No  Yes | 5846(88.7%)  747(11.3%) |  | 4920(93.4%)  347(06.6%) | 926(69.8%)  400(30.2%) | 0.639 |
| Dyslipidemia  No  Yes | 6074(92.1%)  519(07.9%) |  | 5079(96.4%)  188(03.6%) | 995(75.0%)  331(25.0%) | 0.643 |
| **Clinical AJCC staging**  I  II  III  IVa  IVb  IVc | 1349(20.5%)  1555(23.6%)  822(12.5%)  2157(32.7%)  649(09.8%)  61(00.9%) |  | 1099(20.9%)  1283(24.4%)  632(12.0%)  1713(32.5%)  496(09.4%)  44(00.8%) | 250(18.9%)  272(20.5%)  190(14.3%)  444(33.5%)  153(11.5%)  17(01.3%) | 0.139 |
| **Pathological AJCC staging**  I  II  III  IVa  IVb | 1422(24.9%)  1367(23.9%)  806(14.1%)  1757(30.7%)  369(06.4%) |  | 1136(23.9%)  1119(23.6%)  653(13.8%)  1563(32.9%)  275(05.8%) | 286(29.3%)  248(25.4%)  153(15.7%)  194(19.9%)  94(09.7%) | 0.108 |
| **Treatment**  Operation alone  Operation plus RT/CCRT  RT/CCRT  Others | 3455(52.4%)  2589(39.3%)  332(05.0%)  217(03.3%) |  | 2777(52.7%)  2066(39.2%)  260(05.0%)  164(03.1%) | 678(51.1%)  523(39.5%)  72(05.4%)  53(04.0%) | 0.056 |
| **BMI** (IQR) | 24.4(21.8-27.3) |  | 24.1(21.7-27.0) | 25.3(22.8-28.0) | 0.272 |
| **Lab data** (IQR)  HbA1C  Total cholesterol | 6.1(5.7-7.4)  181(157-209) |  | 5.8(5.5-6.0)  183(158-209) | 7.6(6.8-9.2)  175(148-207) | 1.727  0.106 |
| **Medication**  Statins  No  Yes  Metformin  No  Yes | 5887(89.3%)  706(10.7%)  5747(87.2%)  846(12.8%) |  | 4924(93.5%)  343(06.5%)  5018(95.3%)  249(04.7%) | 963(72.6%)  363(27.4%)  729(55.0%)  597(45.0%) | 0.579  1.054 |

* *p* ≤ 0.05

Abbreviations: AJCC, American Joint Committee on Cancer; BMI, body mass index; CCRT, concurrent chemoradiotherapy; DM, diabetes mellitus; IQR, interquartile range; OSCC, oral squamous cell carcinoma; RT, radiotherapy; SMD, standardized mean difference
