# Supplementary material for: Effect of glycated hemoglobin A1c on the survival of patients with oral squamous cell carcinoma: A multi-institutional database cohort study
Source: Front Oncol. 2022 Aug 29;12:952616. doi: 10.3389/fonc.2022.952616 (PMC9465414; doi:10.3389/fonc.2022.952616)
Supplement: Supplementary file 4 [file Table_2.docx]

**Table S2.** Demographic and clinical characteristics of patients within different HbA1c intervals at the initial diagnosis of OSCC.

| **Variables** |  | **Non-DM**  **N=2400** | **HbA1c < 6**  **N=55 *p* value** | | | **6<= HbA1C< 7**  **N=307 *p* value** | | **7<= HbA1C< 8**  **N=299 *p* value** | | **8<= HbA1C< 9**  **N=183 *p* value** | | **HbA1c>=9**  **N=356 *p* value** | |
| --- | --- | --- | --- | --- | --- | --- | --- | --- | --- | --- | --- | --- | --- |
| **Median age at diagnosis**,  years (IQR) |  | 56(50-64) | 60(55-68) | 0.003 | 59(53-66) | | *<0.001 | 58(52-65) | 0.008 | 57(50-63) | 0.673 | 54(48-60) | *<0.001 |
| **Gender**  Female  Male |  | 138(05.8%)  2262(94.2%) | 5(09.1%)  50(90.9%) | 0.249 | 21(06.8%)  286(93.2%) | | 0.444 | 13(04.3%)  286(95.7%) | 0.320 | 8(04.4%)  175(95.6%) | 0.436 | 22(06.2%)  334(93.8%) | 0.746 |
| **Tumor sites**  Lip  Oral tongue  Upper/lower Gum  Floor of mouth  Buccal mucosa  Hard palate  Retromolar trigone |  | 110(04.6%)  746(31.1%)  432(18.0%)  64(02.7%)  886(36.9%)  36(01.5%)  126(05.2%) | 3(05.4%)  19(34.6%)  9(16.4%)  2(03.6%)  19(34.6%)  1(01.8%)  2(03.6%) | 0.943 | 13(04.2%)  120(39.1%)  53(17.3%)  9(02.9%)  97(31.6%)  3(01.0%)  12(03.9%) | | 0.159 | 12(04.0%)  76(25.4%)  66(22.1%)  5(01.7%)  125(41.8%)  4(01.3%)  11(03.7%) | 0.150 | 12(06.6%)  48(26.2%)  33(18.0%)  6(03.3%)  66(36.1%)  2(01.1%)  16(08.7%) | 0.326 | 15(04.2%)  110(30.9%)  55(15.5%)  10(02.8%)  136(38.2%)  8(02.2%)  22(06.2%) | 0.811 |
| **Lifestyle Risk Factors** |  |  |  |  |  | |  |  |  |  |  |  |  |
| Smoking  No  Yes |  | 1162(48.4%)  1238(51.6%) | 27(49.1%)  28(50.9%) | 0.921 | 109(35.5%)  198(64.5%) | | *<0.001 | 110(36.8%)  189(63.2%) | *<0.001 | 78(42.6%)  105(57.4%) | 0.131 | 163(45.8%)  193(54.2%) | 0.354 |
| Betel nuts consumption  No  Yes |  | 1287(53.6%)  1113(46.4%) | 31(56.4%)  24(43.6%) | 0.687 | 132(43.0%)  175(57.0%) | | *<0.001 | 131(43.8%)  168(56.2%) | *0.001 | 87(47.5%)  96(52.5%) | 0.112 | 172(48.3%)  184(51.7%) | 0.061 |
| Alcoholic beverages  No  Yes |  | 899(37.5%)  1501(62.5%) | 17(30.9%)  38(69.1%) | 0.321 | 65(21.2%)  242(78.8%) | | *<0.001 | 77(25.8%)  222(74.2%) | *<0.001 | 49(26.8%)  134(73.2%) | *0.004 | 119(33.4%)  237(66.6%) | 0.141 |
| **Comorbidities** |  |  |  |  |  | |  |  |  |  |  |  |  |
| Hypertension  No  Yes |  | 2219(92.5%)  181(07.5%) | 15(27.3%)  40(72.7%) | *<0.001 | 175(57.0%)  132(43.0%) | | *<0.001 | 204(68.2%)  95(31.8%) | *<0.001 | 140(76.5%)  43(23.5%) | *<0.001 | 302(84.8%)  54(15.2%) | *<0.001 |
| Dyslipidemia  No  Yes |  | 2302(95.9%)  98(04.1%) | 29(52.7%)  26(47.3%) | *<0.001 | 208(67.8%)  99(32.2%) | | *<0.001 | 221(73.9%)  78(26.1%) | *<0.001 | 140(76.5%)  43(23.5%) | *<0.001 | 302(84.8%)  54(15.2%) | *<0.001 |
| **Clinical AJCC staging**  I  II  III  IVa  IVb  IVc |  | 464(19.3%)  496(20.7%)  314(13.1%)  836(34.8%)  280(11.7%)  10(00.4%) | 11(20.0%)  10(18.2%)  12(21.8%)  21(38.2%)  1(01.8%)  0(00.0%) | 0.145 | 65(21.2%)  78(25.4%)  34(11.1%)  99(32.2%)  30(09.8%)  1(00.3%) | | 0.342 | 66(22.1%)  49(16.4%)  47(15.7%)  99(33.1%)  36(12.0%)  2(00.7%) | 0.364 | 38(20.8%)  34(18.6%)  22(12.0%)  66(36.1%)  23(12.5%)  0(00.0%) | 0.896 | 52(14.6%)  77(21.6%)  42(11.8%)  133(37.4%)  50(14.0%)  2(00.6%) | 0.268 |
| **Pathological AJCC staging**  I  II  III  IVa  IVb |  | 466(21.6%)  463(21.5%)  316(14.6%)  747(34.6%)  167(07.7%) | 13(27.1%)  9(18.8%)  10(20.8%)  15(31.2%)  1(02.1%) | 0.485 | 74(26.5%)  60(21.5%)  28(10.1%)  96(34.4%)  21(07.5%) | | 0.240 | 69(25.1%)  56(20.4%)  29(10.5%)  90(32.7%)  31(11.3%) | 0.083 | 42(25.6%)  31(18.9%)  22(13.4%)  53(32.3%)  16(05.8%) | 0.733 | 67(21.3%)  64(20.3%)  50(15.9%)  114(36.2%)  20(06.3%) | 0.838 |
| **Treatment**  Operation alone  Operation plus RT/CCRT  RT/CCRT  Others |  | 1215(50.6%)  991(41.3%)  123(05.1%)  71(03.0%) | 35(63.6%)  13(23.6%)  4(07.3%)  3(05.5%) | 0.029 | 155(50.5%)  129(42.0%)  15(04.9%)  8(02.6%) | | 0.980 | 154(51.5%)  128(42.8%)  11(03.7%)  6(02.0%) | 0.541 | 88(48.1%)  80(43.7%)  10(05.5%)  5(02.7%) | 0.913 | 169(47.5%)  152(42.7%)  21(05.9%)  14(03.9%) | 0.558 |
| **BMI** (IQR) |  | 24.1(21.6-26.7) | 23.8(21.7-25.9) | 0.498 | 25.8(23.1-28.6) | | *<0.001 | 25.9(23.4-28.7) | *<0.001 | 26.0(23.7-28.4) | *<0.001 | 24.6(21.9-27.5) | 0.073 |
| **Lab data** (IQR)  HbA1C  Total cholesterol |  | 5.8(5.6-6.1)  183(158-208) | 5.6(5.4-5.8)  162(149-182) | *<0.001  *0.002 | 6.7(6.4-6.8)  171(149-198) | | *<0.001  *<0.001 | 7.4(7.2-7.6)  167(145-195) | *<0.001  *<0.001 | 8.3(8.1-8.6)  182(150-209) | *<0.001  0.591 | 10.6(9.6-11.9)  184(156-225) | *<0.001  0.053 |
| **Medication**  Statins  No  Yes  Metformin  No  Yes |  | 2229(92.9%)  171(07.1%)  2299(95.8%)  101(04.2%) | 42(76.4%)  13(23.6%)  38(69.1%)  17(30.9%) | *<0.001  *<0.001 | 218(71.0%)  89(29.0%)  178(58.0%)  129(42.0%) | | *<0.001  *<0.001 | 211(70.6%)  88(29.4%)  167(55.9%)  132(44.1%) | *<0.001  *<0.001 | 126(68.9%)  57(31.2%)  102(55.7%)  81(44.3%) | *<0.001  *<0.001 | 272(76.4%)  84(23.6%)  164(46.1%)  192(53.9%) | *<0.001  *<0.001 |

* *p* ≤ 0.05

Abbreviations: AJCC, American Joint Committee on Cancer; BMI, body mass index; CCRT, concurrent chemoradiotherapy; DM, diabetes mellitus; IQR, interquartile range; OSCC, oral squamous cell carcinoma; RT, radiotherapy
