# Supplementary material for: Effect of glycated hemoglobin A1c on the survival of patients with oral squamous cell carcinoma: A multi-institutional database cohort study
Source: Front Oncol. 2022 Aug 29;12:952616. doi: 10.3389/fonc.2022.952616 (PMC9465414; doi:10.3389/fonc.2022.952616)
Supplement: Supplementary file 5 [file Table_3.docx]

**Table S3.** Demographic and clinical characteristics of DM patients within different mean HbA1c intervals during the whole study period

| **Variables** |  | **HbA1c < 6**  **N=52 *p* value** | | | **6<= HbA1C< 7**  **N=283 *p* value** | | **7<= HbA1C< 8**  **N= 186 (reference)** | | | **8<= HbA1C< 9**  **N=110 *p* value** | | **HbA1c>=9**  **N=68 *p* value** | | |  |
| --- | --- | --- | --- | --- | --- | --- | --- | --- | --- | --- | --- | --- | --- | --- | --- |
| **Median age at diagnosis**,  years (IQR) |  | 59(53-66) | 0.054 | 58(52-65) | | 0.037 | 57(49-63) |  | 56(50-61) | | 0.617 | | 55(51-60) | 0.554 | |
| **Gender**  Female  Male |  | 2(03.85%)  50(96.15%) | 0.649 | 20(07.07%)  263(92.93%) | | 0.039 | 5(02.69%)  181(97.31%) |  | 8(07.27%)  102(92.73%) | | 0.080 | | 6(08.82%)  62(91.18%) | 0.073 | |
| **Tumor sites**  Lip  Oral tongue  Upper/lower Gum  Floor of mouth  Buccal mucosa  Hard palate  Retromolar trigone |  | 3(05.77%)  15(28.85%)  9(17.31%)  0(00.00%)  22(42.31%)  0(00.00%)  3(05.77%) | 0.877 | 12(04.24%)  91(32.16%)  63(22.26%)  9(03.18%)  96(33.92%)  3(01.06%)  9(03.18%) | | 0.370 | 8(04.30%)  53(28.49%)  29(15.59%)  5(02.69%)  83(44.62%)  2(01.08%)  6(03.23%) |  | 9(08.18%)  35(31.82%)  16(14.55%)  3(02.73%)  37(33.64%)  2(01.82%)  8(07.27%) | | 0.301 | | 2(02.94%)  17(25.00%)  8(11.76%)  2(02.94%)  33(48.53%)  (00.00%)  6(08.82%) | 0.586 | |
| **Lifestyle Risk Factors** |  |  |  |  | |  |  |  |  | |  | |  |  | |
| Smoking  No  Yes |  | 16(30.77%)  36(69.23%) | 0.034 | 120(42.40%)  163(57.60%) | | 0.295 | 88(47.31%)  98(52.69%) |  | 48(43.64%)  62(56.36%) | | 0.540 | | 31(45.59%)  37(54.41%) | 0.807 | |
| Betel nuts consumption  No  Yes |  | 19(36.54%)  33(63.46%) | 0.167 | 143(50.53%)  140(49.47%) | | 0.495 | 88(47.31%)  98(52.69%) |  | 59(53.64%)  51(46.36%) | | 0.293 | | 36(52.94%)  32(47.06%) | 0.427 | |
| Alcoholic beverages  No  Yes |  | 8(15.38%)  44(84.62%) | 0.010 | 79(27.92%)  204(72.08%) | | 0.170 | 63(33.87%)  123(66.13%) |  | 33(30.00%)  77(70.00%) | | 0.492 | | 24(35.29%)  44(64.71%) | 0.832 | |
| **Comorbidities** |  |  |  |  | |  |  |  |  | |  | |  |  | |
| Hypertension  No  Yes |  | 20(38.46%)  32(61.54%) | *<0.001 | 153(54.06%)  130(45.94%) | | *<0.001 | 130(69.89%)  56(30.11%) |  | 79(71.82%)  31(28.18%) | | 0.725 | | 45(66.18%)  23(33.82%) | 0.571 | |
| Dyslipidemia  No  Yes |  | 30(57.69%)  22(42.31%) | 0.133 | 180(63.60%)  103(36.40%) | | 0.245 | 128(68.82%)  58(31.18%) |  | 75(68.18%)  35(31.82%) | | 0.909 | | 42(61.76%)  26(38.24%) | 0.290 | |
| **Clinical AJCC staging**  I  II  III  IVa  IVb  IVc |  | 8(15.38%)  7(13.46%)  13(25.00%)  20(38.46%)  4(07.69%)  0(000.0%) | 0.029 | 71(25.09%)  56(19.79%)  31(10.95%)  90(31.80%)  34(12.01%)  1(00.35%) | | 0.592 | 51(27.42%)  43(23.12%)  20(10.75%)  58(31.18%)  14(07.53%)  0(00.00%) |  | 23(20.91%)  23(20.91%)  16(14.55%)  35(31.82%)  13(11.82%)  0(000.0%) | | 0.471 | | 14(20.59%)  22(32.35%)  5(07.35%)  21(30.88%)  5(07.35%)  1(01.47%) | 0.317 | |
| **Pathological AJCC staging**  I  II  III  IVa  IVb |  | 9(18.37%)  9(18.37%)  6(12.24%)  19(38.78%)  6(12.24%) | 0.198 | 74(28.46%)  47(18.08%)  34(13.08%)  83(31.92%)  22(08.46%) | | 0.615 | 62(35.84%)  31(17.92%)  23(13.29%)  45(26.01%)  12(06.94%) |  | 23(23.71%)  25(25.77%)  10(10.31%)  32(32.99%)  7(07.22%) | | 0.146 | | 17(27.42%)  18(29.03%)  10(16.13%)  11(17.74%)  6(09.68%) | 0.319 | |
| **Treatment**  Operation alone  Operation plus RT/CCRT  RT/CCRT  Others |  | 23(44.23%)  26(50.00%)  3(05.77%)  0(00.00%) | 0.035 | 120(42.40%)  147(51.94%)  14(04.95%)  2(00.71%) | | *<0.001 | 111(59.68%)  69(37.10%)  2(01.08%)  4(02.15%) |  | 57(51.82%)  41(37.27%)  8(07.27%)  4(03.64%) | | 0.028 | | 43(63.24%)  21(30.88%)  3(04.41%)  1(01.47%) | 0.297 | |
| **BMI** (IQR) |  | 25.4(22.8-27.9) | 0.906 | 26.1(23.5-28.7) | | 0.199 | 25.4(23.2-28.3) |  | 24.9(21.8-27.1) | | 0.070 | | 24.7(22.9-27.2) | 0.297 | |
| **Lab data** (IQR)  HbA1C  Total cholesterol |  | 6(5.6-6.6)  161.5(148-178) | *<0.001  0.039 | 7.1(6.7-8.0)  174(150-195) | | *<0.001  0.491 | 8.1(7.2-9.6)  177(143-212) |  | 9.25(7.8-10.8)  184(156-216) | | *<0.001  0.362 | | 10.75(8.9-12.3)  165(146-220) | *<0.001  0.707 | |
| **Medication**  Statins  No  Yes  Metformin  No  Yes |  | 33(63.46%)  19(36.54%)  29(55.77%)  23(44.23%) | 0.040  *<0.001 | 158(55.83%)  125(44.17%)  93(32.86%)  190(67.14%) | | 0.071  0.002 | 88(47.31%)  98(52.69%)  37(19.89%)  149(80.11%) |  | 60(54.55%)  50(45.45%)  27(24.55%)  83(75.45%) | | 0.229  0.347 | | 42(61.76%)  26(38.24%)  26(38.24%)  42(61.76%) | 0.041  0.003 | |

* *p* ≤ 0.05

Abbreviations: AJCC, American Joint Committee on Cancer; BMI, body mass index; CCRT, concurrent chemoradiotherapy; DM, diabetes mellitus; IQR, interquartile range; RT, radiotherapy
