# Supplementary material for: Effect of glycated hemoglobin A1c on the survival of patients with oral squamous cell carcinoma: A multi-institutional database cohort study
Source: Front Oncol. 2022 Aug 29;12:952616. doi: 10.3389/fonc.2022.952616 (PMC9465414; doi:10.3389/fonc.2022.952616)
Supplement: Supplementary file 6 [file Table_4.docx]

**Table S4.** Modeling for the effects of different HbA1c intervals at the initial diagnosis of OSCC on all-cause mortality and disease-specific mortality compared to those without DM

| **Outcomes** | **HbA1c intervals** | **Crude Hazard Ratio (95% CI)** | **Adjusted Hazard Ratio (95% CI)** | | |  |
| --- | --- | --- | --- | --- | --- | --- |
|  |  |  | **^¶^Model 1** | **^§^Model 2** | **^⁋^Model 3** | **^❡^Model 4** |
| **All-cause mortality** | Non-DM  HbA1c < 6  6 ≤ HbA1c< 7  7 ≤ HbA1c< 8  8 ≤ HbA1c< 9  HbA1c ≥ 9 | 1  *1.66(1.18-2.33)  1.16(0.96-1.39)  1.03(0.85-1.24)  *1.33(1.08-1.65)  1.13(0.95-1.33) | 1  *1.66(1.17-2.33)  1.15(0.96-1.39)  1.06(0.88-1.28)  *1.51(1.22-1.87)  1.17(0.99-1.39) | 1  *1.56(1.08-2.26)  *1.41(1.16-1.71)  *1.25(1.02-1.53)  *1.67(1.33-2.11)  1.13(0.94-1.35) | 1  1.36(0.92-2.03)  *1.56(1.27-1.92)  *1.47(1.18-1.82)  *2.15(1.70-2.74)  *1.47(1.20-1.79) | 1  1.42(0.96-2.10)  *1.59(1.29-1.96)  *1.49(1.20-1.85)  *2.17(1.71-2.76)  *1.47(1.20-1.79) |
| **disease-specific mortality** | Non-DM  HbA1c < 6  6 ≤ HbA1c< 7  7 ≤ HbA1c< 8  8 ≤ HbA1c< 9  HbA1c ≥ 9 | 1  1.33(0.81-2.18)  1.11(0.87-1.41)  1.13(0.89-1.43)  *1.39(1.06-1.83)  *1.29(1.05-1.58) | 1  1.49(0.91-2.46)  1.16(0.91-1.48)  1.15(0.91-1.46)  *1.57(1.19-2.07)  *1.28(1.04-1.58) | 1  1.39(0.81-2.38)  *1.33(1.03-1.72)  *1.37(1.07-1.77)  *1.68(1.25-2.24)  *1.25(1.00-1.56) | 1  1.23(0.70-2.18)  *1.51(1.15-1.99)  *1.69(1.30-2.21)  *2.22(1.62-3.01)  *1.69(1.32-2.16) | 1  1.28(0.73-2.25)  *1.54(1.17-2.02)  *1.69(1.29-2.20)  *2.24(1.65-3.03)  *1.70(1.33-2.17) |

* *p* ≤ 0.05

Abbreviations: CI, confidence interval; DM, diabetes mellitus; OSCC, oral squamous cell carcinoma

^¶^ Model 1 was adjusted for age, sex, tumor site, and clinical AJCC stages of cancer.

^§^Model 2 was adjusted for the variables adjusted in model 1 plus BMI, lifestyle risk factors, and treatment.

^⁋^Model 3 was adjusted for the variables adjusted in model 2 plus comorbidities and medication use.

^❡^Model 4 was built with variables according to the statistical software (a stepwise solution).
