# Supplementary material for: Effect of glycated hemoglobin A1c on the survival of patients with oral squamous cell carcinoma: A multi-institutional database cohort study
Source: Front Oncol. 2022 Aug 29;12:952616. doi: 10.3389/fonc.2022.952616 (PMC9465414; doi:10.3389/fonc.2022.952616)
Supplement: Supplementary file 7 [file Table_5.docx]

**Table S5.** Modeling for the effects of different mean HbA1c intervals during the whole study period on all-cause mortality and disease-specific mortality in OSCC patients with DM

| **Outcomes** | **HbA1c level** | **Crude Hazard Ratio (95% CI)** | **Adjusted Hazard Ratio (95% CI)** | | |  |
| --- | --- | --- | --- | --- | --- | --- |
|  |  |  | **^¶^Model 1** | **^§^Model 2** | **^⁋^Model 3** | **^❡^Model 4** |
| **All-cause mortality** | HbA1c < 6  6 ≤ HbA1c< 7  7 ≤ HbA1c< 8  8 ≤ HbA1c< 9  HbA1c ≥ 9 | *2.12(1.32-3.42)  *1.64(1.20-2.24)  1  *1.68(1.14-2.48)  *1.77(1.15-2.72) | *1.37(1.32-3.42)  *1.69(1.04-2.76)  1  *1.69(1.14-2.51)  *2.13(1.38-3.31) | 1.30(0.92-1.81)  *1.79(1.09-2.94)  1  1.48(0.98-2.24)  *2.02(1.28-3.20) | 1.12(0.79-1.57)  1.33(0.79-2.22)  1  1.31(0.87-1.99)  *1.78(1.12-2.84) | 1.23(0.89-1.70)  1.25(0.76-2.07)  1  1.33(0.89-1.98)  *1.88(1.21-2.92) |
| **disease-specific mortality** | HbA1c < 6  6 ≤ HbA1c< 7  7 ≤ HbA1c< 8  8 ≤ HbA1c< 9  HbA1c ≥ 9 | *1.87(1.02-3.42)  *1.53(1.03-2.28)  1  1.47(0.88-2.44)  1.60(0.91-2.82) | 1.27(0.84-1.90)  1.52(0.83-2.82)  1  1.42(0.85-2.37)  *1.94(1.10-3.43) | 1.25(0.81-1.92)  1.58(0.85-2.95)  1  1.30(0.75-2.23)  *1.98(1.09-3.58) | 1.04(0.68-1.61)  1.10(0.57-2.12)  1  1.19(0.70-2.05)  1.61(0.88-2.96) | 1.16(0.77-1.75)  1.12(0.60-2.11)  1  1.15(0.68-1.94)  1.60(0.90-2.86) |

* *p* ≤ 0.05

Abbreviations: CI, confidence interval; DM, diabetes mellitus; OSCC, oral squamous cell carcinoma

^¶^ Model 1 was adjusted for age, sex, tumor site, and clinical AJCC stages of cancer.

^§^Model 2 was adjusted for the variables adjusted in model 1 plus BMI, lifestyle risk factors, and treatment.

^⁋^Model 3 was adjusted for the variables adjusted in model 2 plus comorbidities and medication use.

^❡^Model 4 was built with variables according to the statistical software (a stepwise solution).
